# Effects of the Probiotic Mixture Slab51® (SivoMixx®) as Food Supplement in Healthy Dogs: Evaluation of Fecal Microbiota, Clinical Parameters and Immune Function

**DOI:** 10.3389/fvets.2020.00613

**Published:** 2020-09-04

**Authors:** Giacomo Rossi, Graziano Pengo, Livio Galosi, Sara Berardi, Adolfo Maria Tambella, Anna Rita Attili, Alessandra Gavazza, Matteo Cerquetella, Albert E. Jergens, Blake C. Guard, Jonathan A. Lidbury, Joerg M. Stainer, Alberto Maria Crovace, Jan S. Suchodolski

**Affiliations:** ^1^School of Biosciences and Veterinary Medicine, University of Camerino, Matelica, Italy; ^2^Clinic ‘St. Antonio’, Madignano, Italy; ^3^College of Veterinary Medicine, Iowa State University, Ames, IA, United States; ^4^Gastrointestinal Laboratory, Texas A&M University, College Station, TX, United States; ^5^Department of Emergency and Organs Transplantation (DETO), Veterinary Clinic Section and Animal Production - Veterinary Hospital, University of Bari “Aldo Moro”, Valenzano, Italy

**Keywords:** healthy dogs, probiotic, microbiota, immunoglobulins, clinical conditions

## Abstract

The gut microbiota plays a crucial role in several physiologic functions of the host. In humans and animals, manipulation of the intestinal microbiota by oral administration of probiotic lactic acid bacteria plays a significant role in modulating the immune system. The aim of this study was to evaluate the safety of the probiotic mixture Slab51® and the capacity of this mixture to stimulate immune function in healthy dogs. Twenty dogs were divided in two groups and received a control diet or the same diet supplemented with a dose of 400 billion cfu of lyophilized bacteria for a period of 60 days. Body weight, food intake, body condition score (BCS), fecal score (FSS), fecal immunoglobulin IgA concentration, plasma IgG concentration, and fecal microbiota composition were monitored. Weight, food intake, BCS, FSS, and biochemical parameters remained unchanged during the treatment in both groups of animals. The fecal microbiota showed a significant decrease in the abundance of *Clostridium perfringens* and a significant increase in the abundance of beneficial Bifidobacterium and Lactobacillus organisms (*p* < 0.05). Fecal IgA and plasma IgG levels were significantly higher in the group receiving the probiotic compared to healthy controls. These data show that dietary supplementation with the probiotic mixture Slab51® is safe and well-tolerated, modulating the composition of the intestinal microbiota, and enhancing specific immune functions in healthy dogs.

## Introduction

The aim of the present study was to perform an open-label pilot trial to assess clinical, immunological, and microbiological effects of the multi-strain probiotic Slab51® (SivoMixx®, Ormendes SA, Jouxtens-Mézery, CH) in healthy dogs. SivoMixx® is a multi-strain product, which contains viable lyophilized bacteria consisting of five strains of lactobacilli, two strains of bifidobacteria, and one strain of *Streptococcus salivarius* subsp. *thermophilus*. We hypothesized that Slab51® administration to healthy dogs would beneficially modulate the fecal microbiota as well as immunological parameters. To treat the dogs in this study, we used high concentrations of live bacteria, although recently excellent effects in terms of performance parameters (body weight and feed conversion) and gastrointestinal health were obtained also using inactivated bacteria (1). Not all growth-promoting effects are mediated by bacterial metabolites or active colonization of the gastrointestinal tract, and inactivated or live probiotics had a similar performance, superior to the other commonly used growth promoter, as zinc bacitracin (1). Similar results and conclusions were reported by other authors, demonstrating any significant differences on the influences of probiotic supplementation on lymphoid organs weights (2). In this optic our approach for this study was to evaluate the impact of live and concentrated probiotic bacteria on systemic and mucosal immune-response in healthy dogs, evaluating directly immunoglobulin concentration. Other studies evidenced that the intestinal microbiota plays a crucial role in host defense as demonstrated by their ability to modulate both innate and acquired immunity at the local and systemic level (3, 4). Due to immunological properties, specific strains of lactic acid bacteria (LAB) that often are contained in a probiotic have raised interest in recent years. When ingested as a feed supplement in sufficient numbers, probiotics are live microorganisms that beneficially affect the gastrointestinal health, going far beyond their conventional nutritional effect (5). The mechanisms underlying the immune modulating properties of probiotics are not fully understood. However, these actions may be due to the ability of probiotics to correct intestinal dysbiosis and/or mediate host responses through a direct adjuvant effect on immune factors, such as cytokines (4). In fact, several strains of LAB were shown to enhance the non-specific immunity *in vitro* as well as *in vivo*, including the release of tumor necrosis factor-α and interleukin 6 (6), increased phagocytosis in mice and humans (7, 8), and to stimulate natural killer cell activity (9, 10). The ability of LAB to specifically modulate the host immune response to pathogens has also been demonstrated (11). An increase in rotavirus-specific antibodies was detected in children with acute rotavirus diarrhea who received *Lactobacillus rhamnosus* (10, 12). Moreover, it was shown that administration of *Lactobacillus johnsonii* to healthy human volunteers boosted the systemic IgA response to the *Salmonella typhi* vaccine Ty21a (13).

Stress or dietary changes can affect the intestinal microbiota of dogs and probiotics might have beneficial effects in these dogs. Important changes of the intestinal microbiota also occur at weaning, and events during this early period of life may have a strong effect on the overall health of the dog throughout their life, in particular on the development of their immune system. Therefore, the rationale for adding probiotics to certain types of pet foods, particularly for puppies, would appear attractive. Our objective was to test the safety and palatability of Slab51®, to assess its capacity to modify the gut microbiota composition, and to stimulate immune function in dogs when added to the dog's standard alimentary regimen.

## Materials and Methods

### Slab51® (SivoMixx®)

Slab51® (SivoMixx®, Ormendes SA, Jouxtens-Mézery, CH) is a commercial multi-strain probiotic containing 200 billion lactic acid bacteria per 1.5 grams of product, comprised of the following strains: *Streptococcus thermophilus* DSM 32245, *Bifidobacterium lactis* DSM 32246, *Bifidobacterium lactis* DSM 32247, *Lactobacillus acidophilus* DSM 32241, *Lactobacillus helveticus* DSM 32242, *Lactobacillus paracasei* DSM 32243, *Lactobacillus plantarum* DSM 32244, and *Lactobacillus brevis* DSM 27961.

### Animals and Diets

Twenty clinically healthy dogs of different breeds were enrolled into the trial (body weight: mean 20.1 kg, range: 18–22.3 kg). Their ages ranged between 2½ and 4 years (mean: 3.1 years). The enrolled dogs and their owners received written information on the trial and all owners gave their written informed consent to participate in the study. All the dogs had been dewormed and vaccinated against rabies, distemper, and hepatitis and had never been exposed to probiotics and antibiotics before the beginning of the trial. Ten dogs each were randomly assigned to either the control group or the test group with equal sex distribution. The test group (Group A) received a commercial, nutritionally complete, extruded dry dog food (Maintenance dry dog food, Nutrix® Castelraimondo, Macerata (MC); moisture 10%, 23% protein, 8.5% fat, 2.5% fiber, 8% ash, 14.2 kJ metabolizable energy/g) supplemented with Slab51®. The probiotic was added to the diet at a dose of 400 billion lyophilized bacteria daily for 60 days. Care was taken to ensure that all dogs consumed an accurate probiotic dosage at each feeding. The dosage was based on previous unpublished studies that demonstrated adequate, albeit transient intestinal colonization in dogs, when administered in an earlier trial. The control group (Group B) received the same dry dog food without any additive. Dogs consumed fresh water *ad libitum* and food was offered for 20 min twice daily. To ensure that administration of the probiotic did not adversely affect food palatability and promoted or maintained the health of the dogs, food intake, body weight, body condition score (BCS, using a scoring system developed by Nestlè Purina), and fecal score (FSS, using a scoring system developed by Nestlè Purina) were controlled regularly, from the start (T0) to the end of the trial (T8).

### Fecal Microbiota Analysis and Measurement of IgA Concentrations in Feces

Fecal samples were collected immediately after a spontaneous evacuation and frozen in liquid nitrogen for microbiota analysis and measurement of IgA concentration. Since Slab51® was administered orally and was expected to act primarily at the mucosal level, secretory IgA was analyzed in the feces.

#### Measurement of IgA in Feces

A small aliquot (0.5 g) of feces from each dog were diluted in 5 ml of PBS and vortexed in a falcon tube. All falcon tubes were centrifuged at 4,000 g for 5 min at 11°C. The supernatants were then collected and frozen at −80°C until measurement of fecal IgA concentrations by ELISA as follows. For measurement of total IgA concentrations, 96-well microtiter plates (Thermo Fischer Scientific, Roskilde, Denmark) were coated overnight at 4°C with 250 ng/well of mouse anti-dog IgA (AbD Serotec, Oxford, UK) in PBS, pH 7.2. Three washes with PBST (PBS + 0.01% Tween 20) were performed between each incubation step. Free binding sites were blocked with PBST containing 1% bovine serum albumin (Sigma-Aldrich, St. Louis, MO, US) for 1 h at room temperature. Triplicate fecal extracts were diluted in PBS and incubated for 3 h at 37°C. ELISA plates were then incubated with the secondary antibody polyclonal goat anti-canine IgA conjugated with horseradish peroxidase (HRP, AbD Serotec, Oxford, UK) diluted 1:10.000 in PBS, for 90 min at room temperature. Finally, the plates were developed with the ABTS (Sigma-Aldrich, St. Louis, Missouri, US), for 30 min at room temperature. Finally, 1% SDS was used as a stop solution. Plates were read with a Multiskan Ascent (LabSystem, Midland, ON, Canada) and, since a monoclonal canine IgA standard was not available, values were expressed as optical densities (OD 450 nm).

#### Measurement of IgG Concentrations in Plasma

To assess the effect of the probiotic on systemic humoral responses, circulating total IgG concentrations were measured in the plasma. Blood was collected by jugular venipuncture into heparinized tubes at week 0, and every 2 weeks up to week 8 of the trial. Plasma was recovered from whole blood after fractionation and the same ELISA described above was used to analyze the total level of IgG in each plasma sample. The total amount of IgG in the plasma was determined using ELISA plates coated with 100 ng/well of rabbit anti-canine IgG (Jackson Immunoresearch, Cambridge, UK). A monoclonal canine IgG (Europa Bioproducts, Cambridge, UK) was used as a standard; values were therefore expressed as g/L of IgG. ELISA plates were revealed with a sheep anti-canine IgG conjugated with HRP (AbD Serotec, Oxford, UK) as the secondary antibody.

#### Fecal Microbiota Analysis

Fecal samples were taken at T0 and T8 from all dogs.

#### DNA Isolation

100 mg of feces were aliquoted into a sterile 1.7 ml tube (Microtube, Sarstedt AG & Co, Nümbrecht, Germany) containing 150 μl of 0.1 mm zirconia-silica beads and 100 μl of 0.5 mm zirconia-silica beads (BioSpec Products Inc., Barlesville, OK, USA). Samples were then homogenized (FastPrep-24, MP Biomedicals, Irvine, CA, USA) for a duration of 1 min at a speed of 4 m/s. DNA was then extracted with the ZR fecal DNA Mini Prep kit following the manufacturer's instructions (Zymo Research, Irvine, CA, USA).

#### Quantitative PCR (qPCR)

To quantify the bacterial genera within the probiotic and also potential enteropathogens of interest on a species level, a panel of five qPCR assays was performed for specific bacterial groups: *Lactobacillus, Bifidobacterium, Streptococcus, Escherichia coli*, and *Clostridium perfringens*. Real-time PCR conditions were carried out as described previously (14).

#### Illumina Sequencing

The V3–V5 region of the 16S rRNA gene was amplified with primers 530F (5′ -GTGCCAGCMGCNGCGG-3′) and 926R (5′ -CCGTCAATTC(A/C)TTTGAGTTT-3′) at the MR DNA Laboratory (Shallowater, TX, USA). A 100 ng (1 μl) aliquot of each DNA sample was used for a 50 μl PCR reaction. HotStarTaq Plus Master Mix Kit (Qiagen, Valencia, CA, USA) was used for PCR under the following conditions: 94°C for 3 min followed by 32 cycles of 94°C for 30 s; 60°C for 40 s and 72°C for 1 min; and a final elongation step at 72°C for 5 min. PCR amplification products were verified on 2% agarose gels and samples were purified using calibrated Ampure XP beads (Agencourt Bioscience Corporation, Danvers, MA, USA). The Nextera® DNA sample Preparation kit including sequencing adapters and sample specific barcodes was used to prepare a DNA library and sequenced at MR DNA on an Illumina MiSeq instrument. Raw sequence data were screened, trimmed, de-noised, filtered, and depleted of chimeras using the QIIME v1.9 (Quantitative Insights Into Microbial Ecology) open-source pipeline (15). Operational taxonomic units (OTUs) were assigned based on at least 97% sequence similarity using QIIME 1.9.

### Biochemical Profile

A biochemical profile consisting of glucose, urea, creatinine, GGT, GOT, AST, ALP, total protein, albumin, γ-globulin, and cholesterol was performed collecting blood from jugular venipuncture into a serum tube, at the start (T0) and at the end (T8) of the study. The biochemical profile was determined using an automated analyzer (BT 3000 Plus, Biotecnica Instruments, Rome, Italy).

### Statistical Analysis

Cardinal data were assessed for normality using Shapiro-Wilk test. Food intake, body weight, and blood biochemical parameters were compared between groups using a *t*-test or a Mann-Whitney test, where appropriate; food intake and body weight were also compared between study times within each group using repeated measures ANOVA or Friedman test, where appropriate; blood biochemical parameters were also compared between the beginning (T0) and the end of the study (T8) within each group using paired *t*-tests or Wilcoxon matched-pairs test, where appropriate.

BCS and FSS were analyzed with Mann-Whitney tests and with Friedman test to perform comparison between groups and between study times within each group, respectively.

Antibody titers in sera and feces were compared between groups with a Student's *t*-test. Within each group an ANOVA for repeated measure followed by a Holm-Sidak *post-hoc* test was used to compare each study time vs. T0.

Data were statistically analyzed with GraphPad Prism, version 8.2.1 for MacOS (GraphPad software Inc., San Diego, California, USA). A difference with a *p*-value < 0.05 was considered statistically significant for all the analyses described above.

The microbiota data were tested for normality using the Shapiro-Wilk test (JMP 10, SAS software Inc.). Because most datasets did not meet the assumptions of normal distribution, comparisons within groups were determined using non-parametric Wilcoxon tests. For taxa summary analysis, taxa that were present in at least 50% of all samples were included in Illumina sequencing data analysis. In addition to this, only taxa that comprised 1% abundance or more on average amongst all groups were included in the analyses. The resulting *p*-values of the Wilcoxon test were adjusted for multiple comparisons using the Benjamini & Hochberg's False Discovery Rate (FDR), and an adjusted *q* < 0.05 was considered statistically significant (16).

## Results

### Clinical Effects

None of the dogs showed any side effects during the trial and remained healthy. Food intake and body weight, BCS, FSS, and serum biochemical parameters (data not shown) did not differ statistically significantly between the two groups during the trial (*p* > 0.05).

### Fecal IgA Titers

**Figure 1:**
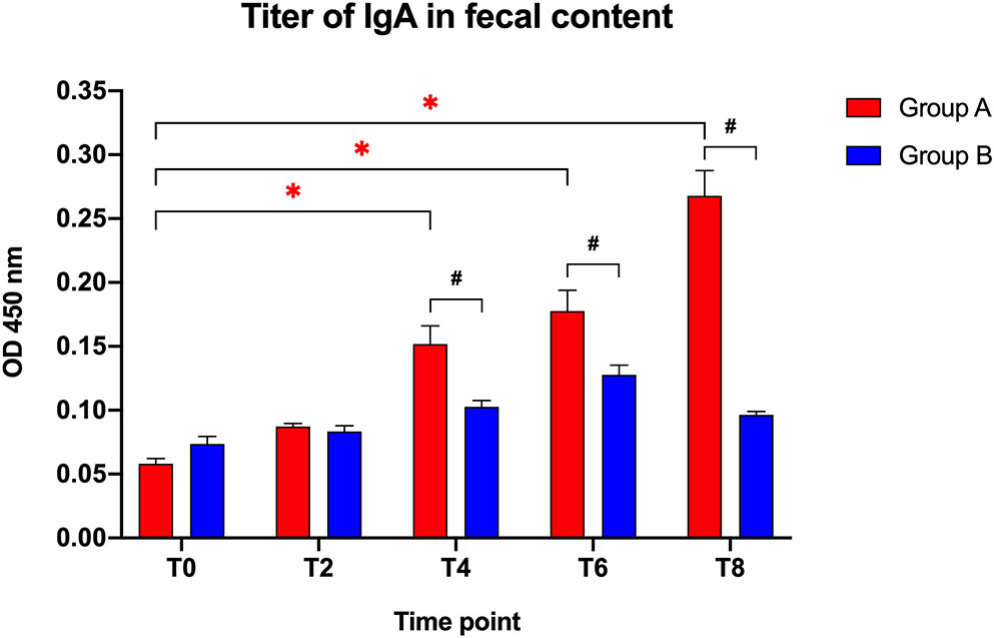
Immunoglobulin IgA titers in fecal contents collected at the beginning of the study (T0), after 2 weeks (T2), 4 weeks (T4), 6 weeks (T6), and 8 weeks (T8) in dogs fed the same diet with (Group A) or without Slab51® probiotic supplement (Group B). Black hashtags indicate statistical significance between groups. Red asterisks indicate statistical significance by study times within group A.

The IgA fecal titers (Figure 1) were not significantly different between the two groups at the beginning of the study (T0: *t* = 2.183; *p* = 0.142) or after 2 weeks (T2: *t* = 0.768; *p* = 0.452), but showed a significantly higher titer in the Slab51®-treated group (Group A) compared to control group (Group B) at T4 (*t* = 3.214; *p* = 0.005), T6 (*t* = 2.796; *p* = 0.012), and T8 (*t* = 8.587; *p* < 0.0001).

When comparing the increase of fecal IgA titers over time compared to baseline (T0), a progressive increase in the antibody titer was observed within Group A (*F* = 71.697; *p* < 0.0001), with a significant difference observed at T4, T6, and T8 (*p* < 0.05 for each). In Group A, the IgA titer at 8 week (0.268 ± 0.020 OD_450nm_ ± SEM) was 4.6-fold higher than the initial titer (0.058 ± 0.004 OD_450nm_ ± SEM).

Group B did not show significant change in fecal antibody titers during the study (*F* = 22.1; *p* > 0.05). Also, the fecal IgA titer at 8 weeks (0.096 ± 0.002 OD_450nm_ ± SEM) was not statistically different (*p* > 0.05) from the IgA titer at baseline (0.074 ± 0.006 OD_450nm_ ± SEM).

### Plasma IgG Titers

**Figure 2:**
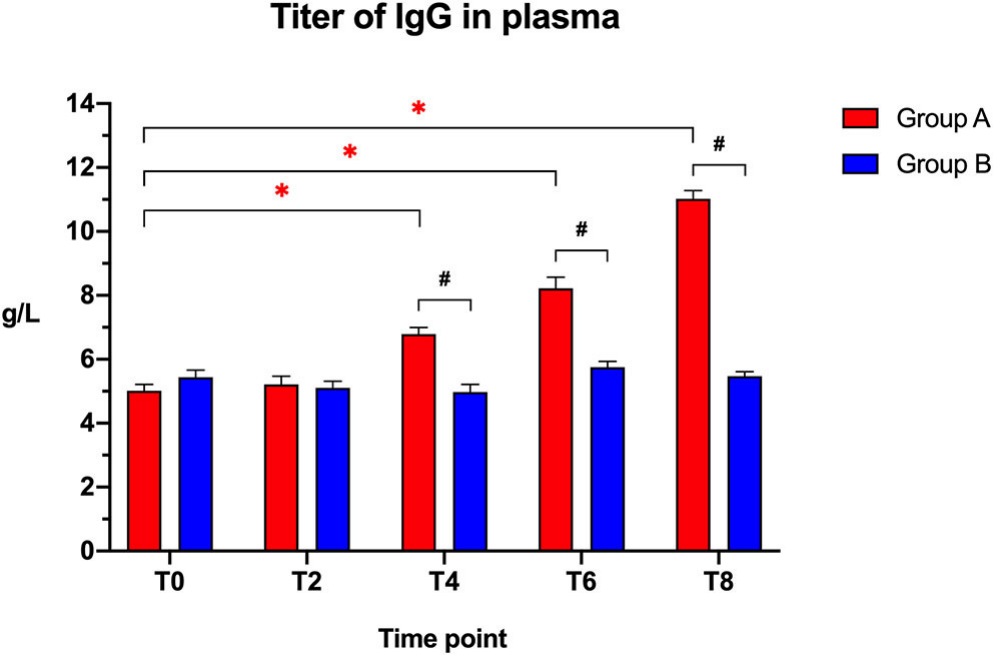
Immunoglobulin IgG titers in the plasma collected at the beginning of the study (T0), after 2 weeks (T2), 4 weeks (T4), 6 weeks (T6), and 8 weeks (T8) in dogs fed the same diet with (Group A) or without (Group B) Slab51® probiotic supplement. Black hashtags indicate statistical significance between groups. Red asterisks indicate statistical significance by study times within group A.

The IgG plasma titers (Figure 2) were similar between the two groups at the beginning of the study (T0: *t* = 1.405; *p* = 0.1771) and after 2 weeks (T2: *t* = 0.3141; *p* = 0.7571), but showed a significantly higher titer in the Slab51®-treated group (Group A) compared to controls (Group B) at T4 (*t* = 5.748; *p* < 0.0001), T6 (*t* = 6.346; *p* < 0.0001), and at T8 (*t* = 18.765; *p* < 0.0001).

When comparing the increase of plasma IgG titer over time compared to baseline (T0), a progressive increase in the antibody titer was observed within Group A (*F* = 161.146; *p* < 0.0001), with a significant increase being observed at T4, T6, and T8 (*p* < 0.05 for each). In Group A, the IgG titer at 8 weeks (11.020 ± 0.261 g/L ± SEM) was 2.2-fold higher than the initial titer (5.012 ± 0.199 g/L ± SEM).

No significant difference in plasma antibody titers was observed within group B when comparing the initial value (T0) to all of the other time points (*F* = 6.242; *p* > 0.05). In Group B, the IgG titer at 8 weeks (5.472 ± 0.138 g/L ± SEM) was not statistically significantly different (*p* > 0.05) from the initial titer (5.435 ± 0.225 g/L ± SEM).

### Gut Microbiota Analysis

**Figure 3:**
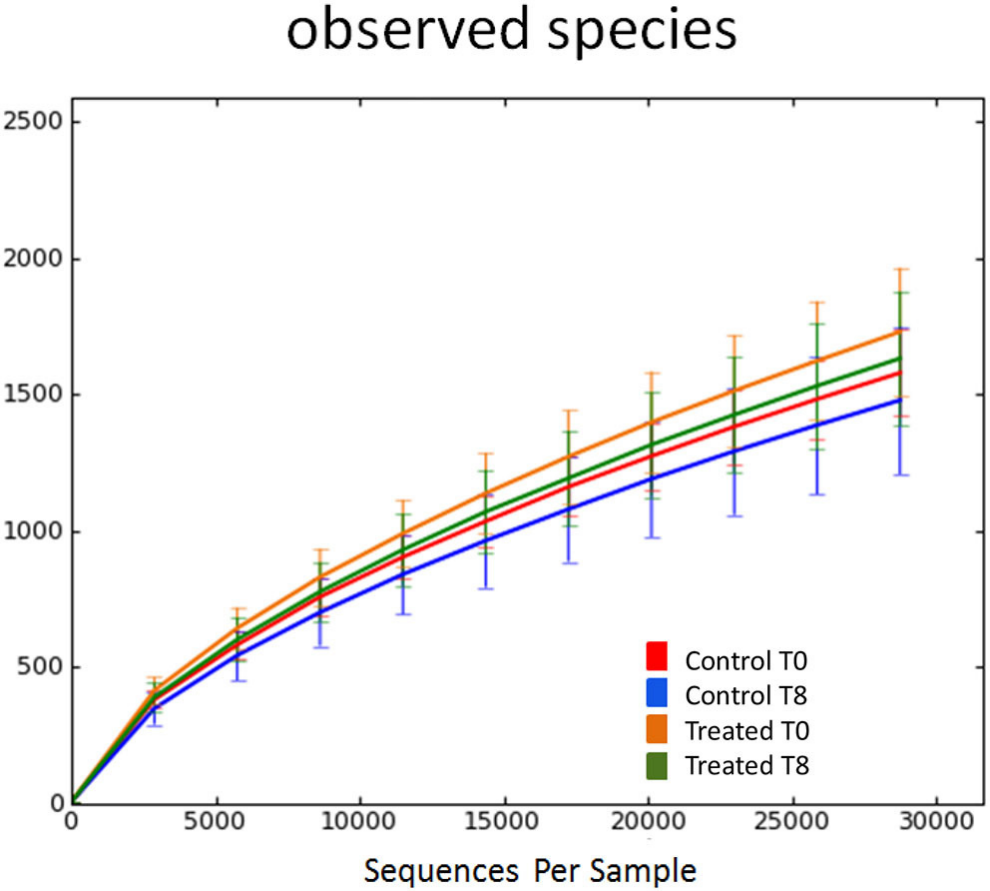
Rarefaction analysis of observed species using 16S rRNA gene sequences obtained from fecal samples from dogs. Lines represent the mean and error bars represent standard deviations. The analysis was performed on a randomly selected subset of 28,754 sequences per sample. Red line = control group (Group B) at T0. Blue line = control group (Group B) at T8. Orange line = treated group (Group A) at T0. Green line = treated group (Group A) at T8.

**Figure 4:**
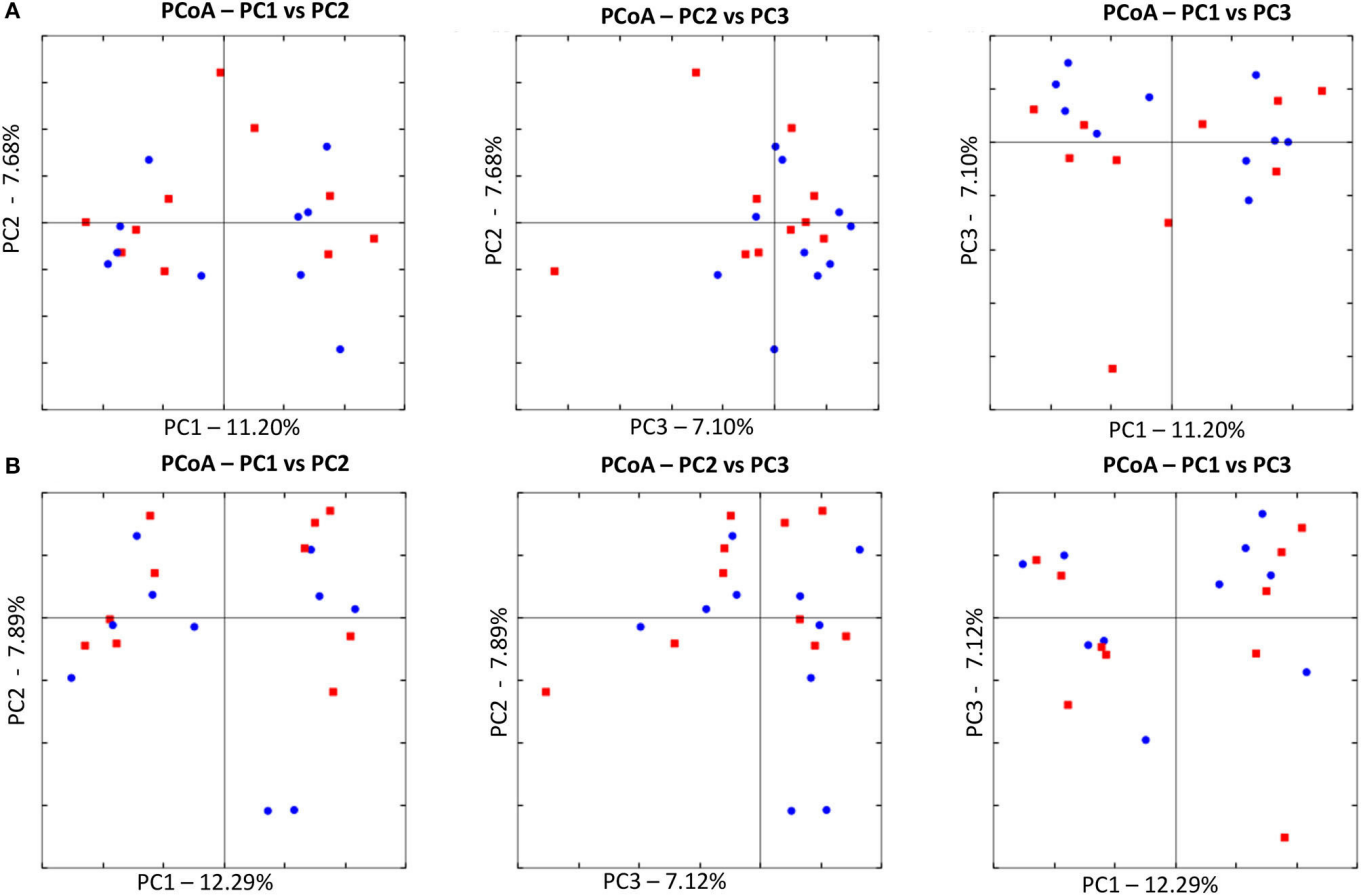
Principal Coordinate Analysis (PCoA) of unweighted UniFrac distances of 16S rRNA genes. **(A)** The control group at T0 (red) and at T8 (blue). There was no difference in clustering between the two time points, indicating no significant shift in microbiota composition (ANOSIM; *p* = 0.8630). **(B)** The treated at T0 (red) and at T8 (blue). There was no difference in clustering between the two time points, indicating no significant shift in microbiota composition (ANOSIM; *p* = 0.4210).

**Figure 5:**
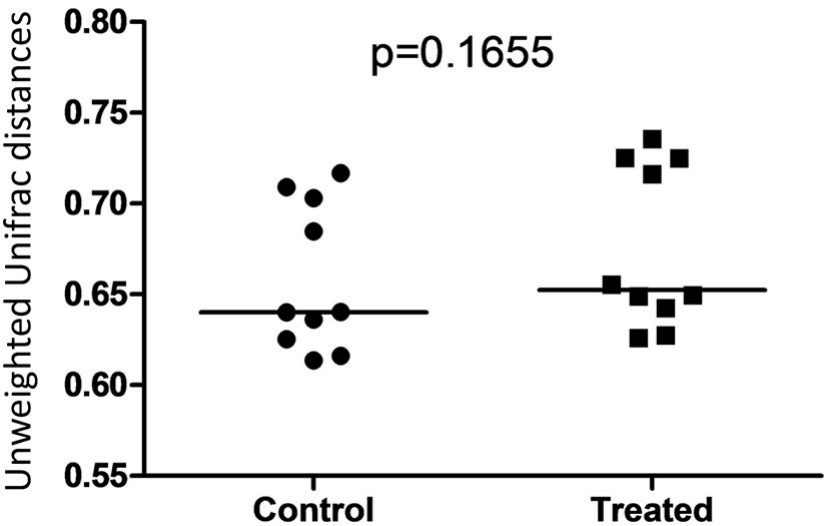
Dot plot representing unweighted UniFrac distances between T0 and T8 in control and treated dogs. There was no significant difference between distances associated with microbial communities in control (Group B) and treated (Group A) dogs.

**Figure 6:**
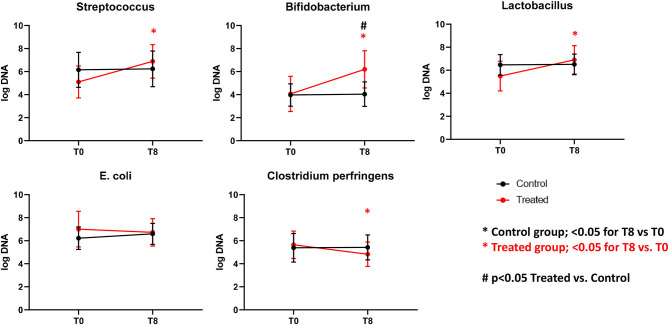
Graphs representing qPCR performed on select bacterial groups. Control (Group B), Treatment (Group A). *Significant difference between T0 and T8 (black = control group, red = treated group). #Significant difference between treated and control group and indicated time point.

Illumina sequencing analysis yielded 2,066,814 quality sequences for the 40 samples analyzed (mean ± SD = 51,670 ± 10,270). There were no significant differences between groups when comparing alpha diversity measures (Figure 3, Table 1). Also, there were no significant differences between microbial communities or unweighted UniFrac distances found amongst all groups (Figures 4, 5). Univariate statistics based on specific bacterial abundances obtained from sequencing results revealed no significant differences between groups (Table 2). Quantitative PCR was performed on select bacterial groups that were either underrepresented in sequencing data or of particular interest in this study (Figure 6). Statistical analysis (Table 3) of this data revealed that *Streptococcus* and *Bifidobacterium* were significantly increased in dogs in the treated group B at T8 compared to T0 (*p* = 0.0008 and 0.0001, respectively), and *C. perfringens* was significantly decreased at T8 vs. T0 (*p* = 0.0206).

**Table 1:** Summary of alpha diversity measures for the fecal microbiota in dogs.

	**(Mean ± SD)**						
	**Control (B) T0**	**Control (B) T8**	**Treated (A) T0**	**Treated (A) T8**	**Baseline (A vs. B) *p*-value**	**Control (B) (T0 vs. T8) *p*-value**	**Treated (A) (T0 vs. T8) *p*-value**
Shannon	5.87 ± 0.43	5.48 ± 0.86	6.24 ± 0.60	5.90 ± 0.70	0.1620	0.3075	0.3075
Observed species	1579.30 ± 158.12	1479.00 ± 271.13	1729.90 ± 234.93	1632.10 ± 245.42	0.1303	0.4727	0.3845
Chao1	3564.41 ± 706.55	3464.37 ± 789.11	3874.96 ± 610.33	3760.09 ± 713.32	0.3847	0.9698	0.6232

**Table 2:** Taxa summary for 16S rRNA gene sequencing data.

	Median (min–max) *in percent						
**Taxa**	**Control B T0**	**Control B T8**	**Treated A T0**	**Treated A T8**	**Baseline (control vs. treated) *q*-value**	**Control (T0 vs. T8) *q*-value**	**Treated (T0 vs. T8) *q*-value**
**Phylum**							
Actinobacteria	2.29 (1.11–13.25)	4.99 (2.03–12.03)	5.11 (0.29–9.88)	7.08 (1.80–15.58)	0.6303	0.1514	0.2123
Bacteroidetes	10.25 (0.27–24.65)	3.63 (0.19–18.73)	12.17 (0.15–32.28)	3.69 (0.73–16.36)	0.5205	0.2780	0.2560
Firmicutes	83.16 (67.10–96.19)	86.68 (73.35–94.35)	71.42 (47.67–88.02)	83.62 (74.74–90.32)	0.6480	0.3217	0.1280
Fusobacteria	0.85 (0.13–9.57)	0.73 (0.15–8.10)	2.62 (0.30–26.34)	0.56 (0.19–14.76)	0.6150	0.8501	0.2476
**Class**							
Coriobacteriia	2.22 (0.43–12.74)	4.02 (1.93–11.86)	4.17 (0.24–9.85)	4.14 (1.73–13.08)	1.0000	0.2271	0.5771
Bacteroidia	10.25 (0.27–24.65)	3.63 (0.19–18.73)	12.17 (0.15–32.28)	3.69 (0.73–16.36)	1.0000	0.4170	0.1920
Bacilli	4.87 (1.13–21.83)	4.52 (0.58–61.01)	2.74 (1.24–16.21)	2.87 (0.85–37.16)	0.9097	1.0000	0.5672
Clostridia	62.97 (11.22–84.11)	60.39 (17.80–90.34)	63.98 (43.83–83.63)	63.92 (43.05–80.31)	0.8804	0.9698	0.7913
Erysipelotrichi	11.34 (1.04–49.99)	9.82 (0.68–32.30)	3.27 (0.79–9.44)	6.57 (1.27–39.64)	0.1542	1.0000	0.2712
Fusobacteriia	0.85 (0.13–9.57)	0.73 (0.15–8.10)	2.62 (0.30–26.34)	0.56 (0.19–14.76)	0.9225	1.0000	0.3714
**Order**							
Coriobacteriales	2.22 (0.43–12.74)	4.02 (1.93–11.86)	4.17 (0.24–9.85)	4.14 (1.73–13.08)	1.0000	0.2271	0.5771
Bacteroidales	10.25 (0.27–24.65)	3.63 (0.19–18.73)	12.17 (0.15–32.28)	3.69 (0.73–16.36)	1.0000	0.4170	0.1920
Lactobacillales	3.69 (0.73–21.57)	2.70 (0.53–60.92)	2.66 (0.79–15.51)	2.69 (0.66–37.10)	0.8501	1.0000	0.8501
Clostridiales	62.97 (11.22–84.11)	60.39 (17.80–90.34)	63.97 (43.83–83.63)	63.92 (43.05–80.31)	0.8804	0.9698	0.9496
Erysipelotrichales	11.34 (1.04–49.99)	9.82 (0.68–32.30)	3.27 (0.79–9.44)	6.57 (1.27–39.64)	0.1542	1.0000	0.2712
Fusobacteriales	0.85 (0.13–9.57)	0.73 (0.15–8.10)	2.62 (0.30–26.34)	0.56 (0.19–14.76)	0.9225	1.0000	0.3714
**Family**							
Coriobacteriaceae	2.22 (0.43–12.74)	4.02 (1.93–11.86)	4.17 (0.24–9.85)	4.14 (1.73–13.08)	1.0000	0.7570	0.7694
Bacteroidaceae	5.76 (0.10–22.92)	0.39 (0.13–5.79)	4.85 (0.10–28.98)	1.99 (0.31–6.33)	0.9171	0.6060	0.3785
Streptococcaceae	0.83 (0.31–7.72)	1.23 (0.25–55.72)	1.44 (0.26–8.58)	1.54 (0.34–35.87)	0.7913	1.0000	0.8618
Clostridiales|f__	2.20 (0.61–8.48)	1.59 (0.44–2.98)	3.03 (1.43–6.08)	3.09 (1.19–4.35)	0.8100	1.0000	0.9171
Clostridiaceae	27.59 (3.28–45.51)	22.81 (4.79–43.12)	26.95 (11.35–36.68)	21.90 (5.79–38.35)	1.0000	0.9097	0.9698
	Median (min–max) *in percent						
**Taxa**	**Control T0**	**Control T8**	**Treated T0**	**Treated T8**	**Baseline (control vs. treated)** ***q*****-value**	**Control (T0 vs. T8)** ***q*****-value**	**Treated (T0 vs. T8)** ***q*****-value**
Lachnospiraceae	20.19 (3.11–46.57)	19.58 (4.47–30.20)	22.58 (10.93–49.12)	25.95 (11.93–48.20)	1.0000	1.0000	0.7878
Peptococcaceae	1.79 (0.08–3.36)	1.92 (0.07–5.01)	1.14 (0.08–5.57)	1.77 (0.14–4.91)	0.8152	1.0000	0.8152
Ruminococcaceae	1.83 (1.07–6.52)	2.18 (0.15–8.01)	3.92 (0.11–8.55)	2.70 (0.26–9.68)	0.6197	0.9776	0.7436
Erysipelotrichaceae	11.34 (1.04–49.99)	9.82 (0.68–32.30)	3.27 (0.79–9.44)	6.57 (1.27–39.64)	0.2570	1.0000	0.4520
Fusobacteriaceae	0.85 (0.13–9.57)	0.73 (0.15–8.10)	2.62 (0.30–26.34)	0.56 (0.19–14.76)	0.7688	1.0000	0.6190
**Genus**							
*Collinsella*	1.82 (0.16–9.15)	3.79 (1.62–11.60)	3.74 (0.22–9.29)	3.68 (1.66–12.21)	1.0000	0.9841	0.7144
*Bacteroides*	5.76 (0.10–22.92)	0.39 (0.13–5.79)	4.85 (0.10–28.98)	1.99 (0.31–6.33)	1.0000	0.7878	0.4921
*Streptococcus*	0.79 (0.26–7.71)	0.79 (0.25–55.69)	1.24 (0.24–8.56)	1.16 (0.30–35.85)	0.9910	1.0000	0.8962
Clostridiales|f__|g__	2.20 (0.61–8.48)	1.59 (0.44–2.98)	3.03 (1.43–6.08)	3.09 (1.19–4.35)	1.0000	1.0000	0.9538
Clostridiaceae|g__1	2.78 (0.37–11.38)	1.72 (0.77–11.89)	2.06 (0.72–9.18)	1.50 (0.63–10.89)	1.0000	1.0000	0.9097
Clostridiaceae|g__1	21.57 (0.86–41.47)	21.00 (3.72–37.44)	19.67 (10.33–33.92)	20.13 (4.92–34.22)	0.8466	0.7020	0.9855
Lachnospiraceae|g	3.75 (0.78–7.16)	3.48 (0.47–10.40)	4.23 (1.70–6.44)	5.01 (0.47–11.54)	1.0000	0.9855	0.7469
*Blautia*	8.27 (1.43–26.52)	9.51 (2.18–22.13)	9.07 (1.00–27.99)	12.69 (9.28–25.00)	1.0000	0.9698	0.6900
*Dorea*	2.86 (0.53–8.21)	2.82 (0.23–5.80)	3.99 (0.86–8.04)	2.35 (0.59–14.80)	0.9698	1.0000	0.8458
*Peptococcus*	1.79 (0.08–3.36)	1.92 (0.07–5.01)	1.14 (0.08–5.57)	1.77 (0.14–4.91)	1.0000	1.0000	0.8671
Ruminococcaceae|g__	0.94 (0.53–2.19)	0.75 (0.08–3.29)	1.72 (0.05–5.01)	1.31 (0.11–6.68)	1.0000	1.0000	0.8245
Erysipelotrichaceae|g__	2.21 (0.26–32.30)	2.84 (0.37–15.33)	0.61 (0.27–3.92)	3.36 (0.39–28.07)	0.9200	1.0000	0.8320
Fusobacteriaceae|g__	0.82 (0.09–9.28)	0.69 (0.13–7.52)	2.56 (0.28–25.77)	0.52 (0.17–14.34)	0.8873	1.0000	0.7020
**Species**							
*Collinsella*|*stercoris*	1.44 (0.12–3.13)	2.41 (1.14–3.71)	1.55 (0.10–6.18)	2.82 (1.30–6.76)	1.0000	0.2743	0.5520
Bacteroides|s__	4.13 (0.08–21.15)	0.30 (0.10–5.39)	3.19 (0.09–27.25)	1.25 (0.29–5.58)	1.0000	0.9200	0.7020
Clostridiales|f__|g__|s__	2.20 (0.61–8.48)	1.59 (0.44–2.98)	3.03 (1.43–6.08)	3.09 (1.19–4.35)	1.0000	1.0000	0.9538
Clostridiaceae|g__|s__1	2.78 (0.37–11.38)	1.72 (0.77–11.89)	2.06 (0.72–9.18)	1.50 (0.63–10.89)	1.0000	0.9855	0.9097
Clostridiaceae|g__|s__2	21.57 (0.86–41.47)	21.00 (3.72–37.44)	19.67 (10.33–33.92)	20.13 (4.92–34.22)	0.8466	1.0000	0.9855
Lachnospiraceae|g__|s__	3.75 (0.78–7.16)	3.48 (0.47–10.40)	4.23 (1.70–6.44)	5.01 (0.47–11.54)	1.0000	0.9097	0.7469
*Blautia*|s__	6.62 (1.05–23.26)	5.70 (1.08–20.93)	7.87 (0.37–21.88)	8.60 (6.18–24.33)	1.0000	0.9788	0.8779
*Blautia*|*producta*	2.27 (0.36–3.20)	2.53 (0.57–4.86)	1.91 (0.57–6.01)	3.56 (0.58–5.68)	0.9665	0.9260	0.7872
*Dorea*|s__	2.86 (0.53–8.21)	2.82 (0.23–5.80)	3.99 (0.86–8.04)	2.35 (0.59–14.80)	0.9698	0.8779	0.8458
*Peptococcus*|s__	1.79 (0.08–3.36)	1.92 (0.07–5.01)	1.14 (0.08–5.57)	1.77 (0.14–4.91)	1.0000	0.9695	0.8671
Ruminococcaceae|g__|s__	0.94 (0.53–2.19)	0.75 (0.08–3.29)	1.72 (0.05–5.01)	1.31 (0.11–6.68)	1.0000	1.0000	0.8245
*Eubacterium*|*biforme*	1.00 (0.14–13.71)	1.97 (0.17–15.28)	0.30 (0.10–3.77)	1.88 (0.21–27.95)	0.7020	0.8809	0.5876
*Fusobacterium*|s__	0.82 (0.09–9.28)	0.69 (0.13–7.52)	2.56 (0.28–25.77)	0.52 (0.17–14.34)	0.8873	1.0000	0.5265

**Table 3:** qPCR results for selected bacterial taxa.

	Median (min-max) *log DNA						
	**Control (B) T0**	**Control (B) T8**	**Treated (A) T0**	**Treated (A) T8**	**T8 (B vs. A) *p*-value**	**Control (B) (T0 vs. T8) *p*-value**	**Treated (A) (T0 vs. T8) *p*-value**
*Bifidobacterium*	3.36 (3.20–5.52)	3.46 (3.20–5.82)	3.20 (3.20–8.05)	6.80 (3.20–7.54)	**0.0017**	0.9769	**0.0001**
*Clostridium. perfringens*	5.51 (3.64–7.48)	5.29 (3.64–7.35)	5.83 (3.64–7.15)	4.62 (3.64–6.47)	0.4435	0.9913	**0.0206**
*Escherichia coli*	6.22 (4.65–7.78)	6.18 (5.68–8.40)	7.30 (4.72–8.87)	7.05 (4.47–8.35)	0.9581	0.5558	0.7211
*Lactobacillus*	6.70 (5.00–7.93)	6.44 (5.45–7.56)	5.41 (3.68–7.54)	7.21 (3.68–7.88)	0.6698	0.9954	**0.0149**
*Streptococcus*	6.03 (3.80–8.49)	6.23 (3.80–8.71)	4.61 (3.80–7.86)	7.33 (4.66–8.27)	0.5544	0.9739	**0.0008**

*Bold values are used when p-value is under 0.05 (meaning: significant value)*.

## Discussion and Conclusions

The results of this trial suggest that supplementation of dry food with Slab51 does not have any measurable adverse effect on dogs (Group A), because there were no differences in food intake, weight loss, or serum biochemistry values between the two groups. Interestingly, the trial showed that supplementation with the probiotic Slab51® enhances mucosal (i.e., fecal IgA titer) as well as systemic (i.e., plasma IgG titer) immune parameters in healthy dogs. In addition, evaluation of the intestinal microbiota revealed no significant differences between groups when comparing alpha diversity measures. Also, there were no significant differences between microbial communities or unweighted UniFrac distances found amongst Groups A and B. Univariate statistics based on specific bacterial abundances obtained from sequencing results revealed some interesting differences in fecal microbiota composition. We observed a significant decrease in the abundance of some potentially pathogen species, such as *C. perfringens* but a substantial but insignificant reduction of fecal *E. coli* was also observed. At the same time, a significant increase in the abundances of *Bifidobacterium* spp. and *Lactobacillus* spp. (i.e., species that are part of the beneficial microbiota) (*p* < 0.05) was observed in dogs supplemented with the Slab51® probiotic mixture. Biourge et al. (17) reported about feeding a dry dog food containing a Bacillus strain without any recognizable health benefits. In contrast, recent studies show that a probiotic based on strains of *Bifidobacterium* and *Lactobacillus* has been effective in modulating the presence of some commensal members of the intestinal microbiota, such as various *Clostridial* species, for example reducing some saprophytic but potentially pathogenic species such as *C. perfringens* (18). *C. perfringens* and their toxins in fact can causes diarrhea in both humans and animals (19). Depending on the toxigenic type, *C. perfringens* may also cause other disorders in the intestinal tract, such as food poisoning or necrotic enteritis, in addition to tissue infections accompanied by myonecrosis, such as gas gangrene due to trauma (19). In addition, recent reports demonstrate a substantial reduction of intestinal cells being colonized by enteroinvasive *E. coli* (EIEC), after exposure of cell monolayers to live *Streptococcus*/*Lactobacillus* strains, but not heat-inactivated ones, demonstrating a direct beneficial effect of these probiotics in maintaining intestinal epithelial barrier integrity, by preserving (actin, ZO-1) or enhancing (actinin, occludin) cytoskeletal and tight junctional protein phosphorylation (20). Kanasugi et al. (21) demonstrated an immune stimulation induced by oral administration of a heat-killed *Enterococcus faecalis* (FK-23), stimulating non-specific immune responses in healthy dogs. As was the case in our trial, in this study probiotics were administered orally, primarily targeting the gut-associated lymphoid tissue (GALT). In a similar study (22), in which young-growing healthy puppies were administered dry dog food containing *Enterococcus faecium* SF68, the authors demonstrated an immune stimulation with total fecal IgA concentrations progressively increasing in the group receiving SF68 compared to the control group. As in that study (22), our results suggest a mucosal adjuvant effect of the orally administered Slab51®. We hypothesized that the different probiotic bacterial strains composing Slab51® directly triggered and stimulated the local immune system that is associated with the intestinal mucosa. This is not surprising because it has been demonstrated that commensals are able to trigger a self-limiting humoral mucosal immune response in monoassociated germ-free mice (23). Furthermore, it has been demonstrated recently that mucosal dendritic cells express tight junction proteins and penetrate the gut epithelial monolayer to sample bacteria directly in the intestinal lumen (24). Secretory IgA in the intestine is the most important protective humoral immune factor at this mucosal site (25). It promotes antigen exclusion by inhibiting microbial adherence, colonization and penetration, as well as decreasing food antigen uptake (26, 27). Increased concentrations of total IgG in the plasma indicate also a systemic response to GALT polarization and stimulation by the enteric probiotic microbiota. Outside of the gut, probiotics influence immunoglobulin levels by altering systemic Ig isotope profiles. Oral administration of *L. johnsonii* NCC533 skewed systemic IgG isotypes toward a greater proportion of IgG1, an isotype that is associated with IL-4 induction of B cells and a Th2 predominant immune response (28). In contrast, *L. paracasei* NCC 2461 induced a greater proportion of IgG2a, which resulted from IFNg stimulation of B cells, and is associated with a Th1 predominant immune response (29). Differences in immunoglobulin induction patterns indicate that different probiotic strains can induce unique systemic T-cell responses (28, 29). This could reflect an IgG switch of mucosally primed B cells (30–32), especially of GALT, and may be associated with the increase in mucosal IgA response that likely results from the specific homing of the IgA-producing B cells in the gut (33, 34). Indeed, it was shown that dendritic cell maturation can be induced by probiotics *in vitro*, as characterized by increased expression of MHC II (35). This study also demonstrated that the overall microbiota beta diversity was not significantly changed after intake of Slab51®. Meanwhile, quantitative PCR performed on selected bacterial groups of particular interest in this study, revealed that *Streptococcus* and *Bifidobacterium* were significantly increased in dogs in the group treated with Slab51® at T8 (after 60 days of supplementation) compared to dogs of the same group but at the start (T0) of the study (*q* = 0.0325 and 0.0390, respectively). This suggests that the increased intestinal abundance of some probiotic species such as *Streptococcus* and *Bifidobacterium* could be at the basis of the immune stimulating properties of Slab51® these proprieties were probably not elicited indirectly via modulation of the endogenous microbiota, because the substantial identity of the microbiota composition at T0 and T8 in the Slab51® treated group, but rather directly via an immunoadjuvant mechanism induced by some species with an increased abundance. In conclusion, the results reported here, support the safety and palatability of the probiotic mixture Slab51®, demonstrated by unaltered blood and plasma biochemical parameters in both study groups. Also, dogs supplemented with Slab52 showed no significant differences between microbial communities, found amongst all groups of resident bacteria, but with the only exception for an increased abundance of the probiotic genera *Streptococcus* and *Bifidobacterium* only in the treated group at T8. An interesting adjuvant effect of Slab51® probiotic bacteria at both the mucosal and systemic level in treated dogs, was evident after 2 months of oral supplementation. This effect could be relevant for improving protective immune responses against various infections during the critical weaning period as well as during later stages in life. This study demonstrates that a high concentrated and live probiotic bacteria can significantly enhance the total fecal IgA, and the IgG humoral and systemic immune response following the probiotic challenge. An increase in intestinal IgA can be beneficial to prevent the entry or colonization of enteropathogens, by immune exclusion. However, subsequent studies will need to address the specific quantification of IgA immune responses in relation to bacterial clearance.

## Data Availability Statement

The raw data supporting the conclusions of this article will be made available by the authors, without undue reservation.

## Ethics Statement

The animal study was reviewed and approved by Ethics Committee for Veterinary Clinic and Zoothecnical Studies of the Department of Emergency and Organ Transplantation, from the University of Bari, Italy (Certificate of Approval No. 3/2018). Written informed consent was obtained from the owners for the participation of their animals in this study.

## Author Contributions

GR and GP were responsible for the conception of the study. GR, LG, and AT performed data interpretation and wrote the manuscript. LG, SB, AA, and AC performed fecal IgA and IgG determination. AG, MC, and AJ reviewed the manuscript and provided critical suggestions and comments. BG, JL, JMS, and JSS analyzed the fecal microbiota. All authors discussed the results and approved the final manuscript.

## Conflict of Interest

The authors declare that the research was conducted in the absence of any commercial or financial relationships that could be construed as a potential conflict of interest.
